# Effect of feed form and nutrient density on growth performance, blood parameters, and intestinal traits in broiler breeder pullets

**DOI:** 10.1016/j.psj.2023.102700

**Published:** 2023-04-11

**Authors:** Anvar Amoozmehr, Behrouz Dastar, Omid Ashayerizadeh, Reza Mirshekar, Mohammad Reza Abdollahi

**Affiliations:** ⁎ Department of Animal and Poultry Nutrition, Faculty of Animal Science, Gorgan University of Agricultural Sciences and Natural Resources, Shahid Beheshti Ave, Gorgan, Iran; † Monogastric Research Centre, School of Agriculture and Environment, Massey University, New Zealand

**Keywords:** broiler breeder, diet form, fiber source, pellet, crumble

## Abstract

This study was conducted to evaluate the effect of feed form and nutrient density on growth performance, blood parameters, and intestinal traits of broiler breeder pullets during grower (7–19 wk) and pre-breeder (19 weeks to 5% production) periods. A total of 450 female broiler breeder pullets were used in a completely randomized design with a 3 × 2 factorial arrangement including 3 feed forms (mash, crumble, and pellet) and 2 nutrient densities (standard diet with the nutrient requirement of Ross 308 parent stock nutrition specification, and diluted diet by using sunflower hull to have 10% lower nutrient than the standard diet). Five replicates with 15 pullets per replicate were allocated to each of the 6 treatments. Blood samples were collected at 19 wk of age. Egg production reached 5% in the mid of 25 wk. Results showed that pullets fed crumble or pellet diets had greater body weight gain and a lower feed to gain ratio (F:G; *P* < 0.001). Diet dilution led to a decrease in body weight gain while increasing the F:G (*P* < 0.05). Pullets fed the pellet diets had shorter eating times than those fed crumble diets, whereas the longest eating time belonged to pullets fed mash diets (*P* < 0.001). Pullets fed pellet diet had a greater heterophil to lymphocyte (**H/L**) ratio than those fed crumble or mash diets (*P* = 0.007). Diluting the diet led to a decrease in the H/L ratio (*P* = 0.026). Neither feed form nor nutrient density had a significant effect on body weight uniformity, blood glucose and lipid concentrations, liver enzyme activities, and intestinal traits (P > 0.05). It can be concluded that pelleted or crumbled diets with lower nutrient density can be considered in broiler breeder's pullets feeding with no detrimental effect on their performance or health state.

## INTRODUCTION

Mash, crumble and pellet are the common feed forms in the poultry industry that directly affects growth performance, nutrient digestion, intestinal health, and productivity of the birds (Abdollahi et al., 2014; Guzmán et al., 2015b; Mohammadi Ghasem Abadi et al., 2019). Crumble and pellet feeds are prepared by adding steam to the mash feed in the conditioner during the pelleting process. Pelleting reduces the segregation of feed ingredients and promotes growth performance as well as feed efficiency in chicks (Abdollahi et al., 2013, 2018). Pelleted diets promote metabolizable energy and nutrient intake which is beneficial in broiler chickens (Massuquetto et al., 2020) but this circumstance is questionable in broiler breeders because surplus energy intake negatively influences their performance and fertility and increases the susceptibility to fatty liver (Robinson et al., 1993). It should be considered that consuming high dietary energy can lead to overweight in the birds, accompanied by increasing triglyceride levels in the liver and ovary, and disturbance in ovarian function (Chen et al., 2006). Also, feeding egg layer hens with a pelleted diet can lead to the incidence of feather pecking, poor plumage, featherless skin, dermal wounds, cannibalism, poor performance, and high mortality (Rodenburg et al., 2013; Schreiter et al., 2019).

It may seem that feeding pellets to egg and broiler breeder pullets may have a detrimental effect on their welfare, health state, and productive performance, but the elimination of microorganisms during the pelleting process (Boltz, 2019) encourages feed manufacturers to improve their knowledge for precision feeding in the poultry farms. Diet dilution using natural insoluble fiber sources such as sunflower hull may be the solution to reduce the harmful effect of feeding diets in pellet form. van Krimpen and de Jong (2014) found that the source and level of fiber, diet composition, and physiological status of birds are the most important factors that directly affect breeders’ performance. Digesta retention time in the upper part of the gut will increase with the inclusion of insoluble dietary fiber. Also, insoluble fiber can promote the development and function of the gizzard and digestive organs, HCL and bile acid secretion, and amylase activity, leading to better nutrient digestion (Hetland and Svihus, 2001; Hetland et al., 2003). Increasing dietary fiber can inhibit the intestinal adhesion of pathogens and decrease the incidence of enteric diseases (Bjerrum et al., 2005). The inclusion of sunflower hull at 2% has been reported to improve the average daily gain of pullets from 0 to 21 d of age (Guzmán et al., 2015b). Additionally, it was reported that 1% inclusion of a fiber source in the diet of pullets at 13 wk of age improved proteolytic enzyme activity in the pancreas (Yokhana et al., 2016). The gizzard is a critical digestive organ that directly affects nutrient digestion. It was found that applying 4% cereal straw to the diet as a fiber source caused an increased gizzard weight of pullets by 8.7% (Guzmán et al., 2015a). We hypothesized that reducing dietary nutrient density using sunflower hull as an insoluble fiber could reduce the adverse effects of consuming the pellet and crumble feeds in the broiler breeder pullets. Therefore, the goal of the present study was to evaluate the impact of the feed form (mash, crumble, and pellet) and dietary dilution on the performance, blood parameters, and intestinal traits of broiler breeder hens in the grower and pre-layer stages.

## MATERIALS AND METHODS

All the methods applied in the present study were approved by the Animal Ethics Committee of the Animal Science Faculty at Gorgan University of Agricultural Sciences and Natural Resources, under protocol # 98/181/260 FA.

### Birds, Diets, and Housing Management

A total of 450 broiler breeder pullets at 5 wk of age (Ross 308, mean body weight 694.2 ± 6.89 g) were selected and housed in 30 deep litter floor pens covered with wood shaving. During the adaptation period starting at 36 d of age, birds were fed daily 41 g corn-soy mash diet at 7:00 AM according to the management handbook (Aviagen, 2016). After 1 wk of adaptation, they were fed with 1 of 6 dietary treatments up to the end of the experiment at 5% egg production, which was 25 wk of age. The diets were compared in a completely randomized design with 3 × 2 factorial arrangements consisting of 3 feed forms (mash, crumble, pellet) and 2 dietary nutrient densities (standard and diluted diet). The standard diet was formulated according to Ross 308 parent stock nutrition specification (Aviagen, 2016) for the grower (7–19 wk of age) and pre-breeder (19–5% egg production) stages, whereas the diluted diet was prepared by using sunflower hull to have 90% nutrients recommendations (Table 1). Before diet formulation, all ingredients were analyzed according to the standard AOAC (2005) for dry matter by oven-drying (method 930.15), crude protein by Kjeldahl procedure (nitrogen × 6.25; method 990.02), crude fiber by extraction in acid and alkali solution (method 978.10). Neutral detergent fiber and acid detergent fiber were determined as indicated by van Soest et al. (1991). The gross energy value was determined with a Parr adiabatic oxygen bomb calorimeter using thermos chemical benzoic acid as a standard. The amino acid contents were determined using near-infrared reflectance spectroscopy.

**Table 1:** Ingredients and nutrient composition of the experimental diets (as-is basis).

Ingredients, %	Grower period (7–19 wk)		Pre-breeder period (19 wk—5% egg production)	
	Standard diet	Diluted diet	Standard diet	Diluted diet
Corn grain (CP=7.4%)	59.00	50.57	52.43	60.85
Soybean meal (CP=41%)	19.21	15.85	17.42	20.87
Barley (CP=11.9%)	5.00	5.00	3.00	3.00
Wheat (CP=11.8)	5.00	5.00	5.00	5.00
Wheat bran (CP=14.5%)	5.00	5.00	2.94	2.94
Bentonite	2.60	2.60	2.60	2.60
Sunflower hull^1^	-	12.27	12.39	-
_DL_-Methionine, 99%	0.14	0.12	0.10	0.11
_L_-Lysine-HCL, 78%	0.02	0.01	-	-
_L_-Threonine, 99%	0.04	0.03	-	-
Dicalcium phosphate	1.82	1.64	1.31	1.45
Calcium carbonate	1.04	0.83	1.73	2.05
Vitamin premix^2^	0.25	0.25	0.25	0.25
Mineral premix^3^	0.25	0.25	0.25	0.25
Sodium chloride	0.30	0.27	0.27	0.3
Sodium bicarbonate	0.13	0.11	0.11	0.13
Toxin binder	0.20	0.20	0.20	0.20
Nutrient values (%, unless mentioned)				
Metabolizable energy, kcal/kg	2,520	2,800	2,520	2,800
Crude protein	12.85	14.28	13.05	14.5
Calcium	0.81	0.90	1.08	1.20
Available phosphorus	0.38	0.42	0.31	0.35
Sodium	0.15	0.16	0.15	0.16
Digestible lysine	0.55	0.61	0.56	0.62
Digestible methionine	0.31	0.35	0.29	0.33
Digestible methionine + cystine	0.50	0.55	0.49	0.54
Digestible threonine	0.43	0.48	0.41	0.46
Crude fiber	9.05	2.98	8.97	2.85
NDF^4^	19.06	11.52	18.31	10.69
ADF^5^	11.31	4.04	8.50	3.85
Physical Properties				
Shaker sieve				
1 mm>	36	36	21	21
1–2 mm	19	19	27	27
2–3 mm	25	25	27	27
3 mm<	20	20	25	25
Bulk density, kg/m^3^	600	754	600	700
PDI^6^	90.4	92.6	94	92.2
Hardness, kg^7^	4.9	3.8	4.8	4.9

1 Sunflower hull had 92.8% dry matter, 4.8% crude protein, 52.3% crude fiber, 71.3% NDF, 63.1% ADF, and 1,037 Kcal/kg gross energy.

2 Composition per kilogram of product: vitamin A, 2.090.000 UI; vitamin E 7.600 mg; vitamin, B1 475 mg; vitamin, D3 332.500 UI; vitamin, K3 950mg; nicotinic acid, 8.500 mg; vitamin, B12 3.800 mcg; vitamin, B2 1.900 mg; vitamin, B6 950 mg; folic acid, 237.5 mg; biotin, 38 mg; choline, 72.000 mg; pantothenic acid, 3.800 mg.

3 Composition per kilogram of product: copper, 3.000 mg; iron, 15.000 mg; iodine, 300 mg (in salt); manganese, 21.000 mg; selenium, 90 mg; zinc, 19.500 mg.

4 Neutral detergent fiber.

5 Acid detergent fiber.

6 Pellet durability index.

7 Pellet hardness tester.

Birds had free access to clean tap water throughout the experiment. The pullets were reared in 1.5 m × 2 m floor pens. Each pen had a separate trough feeder and 2 bell drinkers. During 7 to 10 wk of age, a 10 cm feeder space was assigned to the birds in each pen, whereas it was 15 cm from 10 wk onward. The average relative humidity of the rearing house was set at around 60% throughout the experiment, and the temperature was controlled according to Ross 308 parent stock management guideline. From the beginning to 23 wk, the lighting program was 8 h light and 16 h dark with a light intensity of 10 lux. Light stimulation was applied gradually at 23 wk of age with increasing light length from 8 to 11 h and light intensity to 50 lux within 3 d. Light length and intensity from 24 wk to 25 wk of age were 12 h and 50 lux, respectively. The first egg of each experimental unit was seen in the mid of 25 wk, and egg production reached 5% at the end of 25 wk.

### Feed Physical Properties

#### Particle Size

The shaker sieve method was applied to assess the particle size distribution of the standard and diluted diets. A set of sieves sized 1mm >, 1 to 2 mm, 3 mm, and 3 mm >were applied to separate feed particles into different-sized fractions. A sample of feed (100 g) in mash form was passed through the sieve on a shaker for 10 min. The amount of particles retained on each sieve was measured by subtracting the weight of the sieve from the final weight of the sieve and sample after shaking.

#### Bulk Density

A sample of the standard and diluted diets was subjected to bulk density analysis using a glass beaker of known volume (250 cm^3^). The weight of the beaker was first determined. Then feed as mash form was poured into the beaker, and the beaker and the sample contained were weighed again. The weight of the sample was then divided by its volume and the aerated bulk density was recorded.

#### Pellet Durability Index

The Holmen durability tester was used to calculate PDI. Briefly, a sample size of 100 g of pellets is transported through tubes with high-velocity air of 68 mbar for 1 min. Pellets were subjected to impact and shear forces. The PDI was calculated based on the remaining whole pellets that were collected and weighed.

#### Hardness

Amandus Kahl's pellet hardness tester was used for monitoring the hardness of the pellet.

### Performance, Body Weight Uniformity, and Time Eating

Birds were weighed individually at 7, 19, and 25 wk of age, and then body weight gain (**BWG**) was calculated for grower (7–19 wk), pre-layer (19 wk—5% egg production), and whole period (7–5% egg production) of the experiment. Birds had roughly 14 percent higher body weight at the beginning of the experiment (748.4 ± 13.24 g) compared to the target body weight (660 g) recommended in Ross 308 management guide (Aviagen, 2016). So, feed allocation was considered 2 percent lower than the recommended value according to the energy content of the diet so that the amount received energy was similar for all pullets. The feed to gain ratio (**F:G**) was calculated by dividing feed allocation to BWG. After weighing all the birds of each pen at 7, 19, 23, and 25 wk of age, the average body weight and standard deviation were calculated. Then the body weight uniformity was calculated according to the method of Jackson et al. (2004) as 100−[(standarddeviation/mean)×100]. On the last day of 7, 19, 23, and 25 wk, the time pullets spent to consume the total supplied feed was recorded. The feeders were filled at 7:00 AM, as always, and 4 technicians monitored the time difference between feeder filling and emptying. One person focused on each of the 6 experimental units for recording eating time.

### Blood Parameters

Blood samples were obtained from a left-wing vein of 1 bird in each pen, which showed the average weight for the pen at 19 wk of age by syringes with 23-gauge needles. After blood sampling, the birds were marked and returned to their pen. Blood samples were poured into plastic tubes and centrifuged at 3,000 *g* for 10 min at room temperature, and then serum was collected and stored at –20°C until the analysis. The blood serums were measured for triglycerides, cholesterol, high-density lipoprotein-cholesterol **(HDL-c**), aspartate transaminase, and alanine transaminase using commercial kits (Pars Azmoon, Tehran, Iran) by spectrophotometric methods. The very-low-density lipoprotein-cholesterol (**VLDL-c**) values were determined by dividing triglyceride values to unit 5. Low-density lipoprotein-cholesterol (**LDL-c**) values were calculated by subtracting the sum of HDL-c and VLDL-c from total cholesterol (Barbarestani et al., 2020). To assess white blood cell differentiation count, blood samples of the birds̓ right wing vein was collected in tubes contained heparin. Blood sample smears were prepared in triplicate glass slides, and Wright-Giemsa solution was applied to dye the slides. One hundred white blood cells were counted under an optic microscope to measure the heterophil (H) and lymphocyte (L) percentages. Then the H/L ratio was calculated as reported by Gross and Siegel (1983).

### Intestinal Length and Weight

At 23 wk of age, the marked birds on blood sampling day were caught and transferred to the slaughter facility. Hens were weighed and then euthanized by CO_2_ gas to measure gastrointestinal fractions. The intestinal segments were opened at the mesenterium, then the digesta were evacuated, and the fractions were washed in neutral-buffered saline and soaked up excess surface humidity with a paper towel. Then the weight and length of the empty intestinal fractions were measured.

### Statistical Analyses

Data were first tested for normality of residual (Shapiro-Wilk test) and homogeneity of variance (Leven test) considering each pen as the experimental unit. Then, data were analyzed in a completely randomized design with a 3 × 2 factorial arrangement including 3 feed forms (mash, crumble, and pellet) and 2 nutrient densities (standard diet and diluted diet) as main effects using the Mixed procedures of SAS software (SAS, 2013) according to the model $Y_{ijk}=\mu +\alpha_{i}+\beta_{j}+{\left(\alpha \beta \right)}_{ij}+e_{ijk}$ where Y_ijk_= dependent variable; μ= general mean; α_i_= effect of feed form; β_j_= effect of nutrient density; αβ_ij_= effect of interaction between feed form and nutrient density; e_ijk_= residual error. Results in tables are presented as least-square means. Significant differences between treatment means were separated by the protected *t* test (*P* < 0.05). One sample t-test also was used for comparing the difference between each treatment with the BWG and F:G target values reported by the parent stock Ross 308 manual (Aviagen, 2016, 2021). Statistical power was calculated using the Proc GLMPOWER procedure of SAS 9.4 (SAS, 2013) for all traits considering error standard deviation and total sample size when the null hypothesis was false. The power calculations were greater than 0.90 for almost all traits.

## RESULTS

### Productive Performance

The interaction between feed form and dietary nutrient density was not significant for BWG and F:G in the grower, pre-layer, and entire period of the experiment (*P* > 0.05, Table 2). The birds fed a crumble or pellet diet had greater BWG and better F:G during the grower period (*P* < 0.01). No significant difference was observed between pullets that were fed different feed forms for BWG and F:G during the pre-layer period (*P* > 0.05). Pullets who were fed the pellet diet had greater BWG and lesser F:G than those who were fed the mash diet and the values for the crumble diet were intermediate of pellet and mash diets at the entire of experiments (*P* = 0.004). Pullets were fed the diluted diet trend to have lesser BWG at the grower (*P* = 0.08) and entire periods of the experiment (*P* = 0.059), whereas no difference was observed at the pre-layer period (*P* > 0.05). Diluting led to pullets having a greater F:G during all periods of the experiment (*P* < 0.01).

**Table 2:** Influence of feed form and nutrient density on productive performance of broiler breeder pullets in different rearing periods.

		Experimental rearing periods								
Feed form	Nutrient density	Grower (7–19 wk)			Pre-layer (19 wk—5% egg production)			Entire (7 wk—5% egg production)		
		BWG^1^ (g/bird)	FA^2^ g/bird)	F:G^3^	BWG^1^ (g/bird)	FA^2^ (g/bird)	F:G^3^	BWG^1^ (g/bird)	FA^2^ (g/bird)	F:G^3^
Mash	Standard diet	1447	5698	3.94*	917	4254	4.69	2363*	9952	4.21^⁎⁎^
Crumble		1534^⁎⁎^	5698	3.72^⁎⁎^	885	4254	4.82	2419*	9952	4.11^⁎⁎^
Pellet		1610^⁎⁎^	5698	3.54^⁎⁎^	895	4254	4.77	2505^⁎⁎^	9952	3.97^⁎⁎^
Mash	Diluted diet	1430	6314	4.42	916	4680	5.13	2346	10994	4.69
Crumble		1520^⁎⁎^	6314	4.16	878	4680	5.33	2398^⁎⁎^	10994	4.59
Pellet		1542^⁎⁎^	6314	4.10*	869	4680	5.39	2411^⁎⁎^	10994	4.56
SEM^4^		22.61	ND	0.06	28.21	ND	0.14	27.19	ND	0.04
Main effect										
Feed form										
Mash		1438^c^	6006	4.18^a^	916	4467	4.91	2355^b^	10473	4.45^a^
Crumble		1527^b^	6006	3.94^b^	881	4467	5.08	2408^a^^,^^b^	10473	4.35^a^^,^^b^
Pellet		1576^a^	6006	3.82^b^	882	4467	5.08	2458^a^	10473	4.26^b^
SEM^4^		15.98	ND	0.04	19.94	ND	0.10	19.23	ND	0.03
Nutrient density										
Standard diet		1530	5698	3.73^b^	899	4254	4.76^b^	2429	9952	4.10^b^
Diluted diet		1497	6314	4.22^a^	888	4680	5.29^a^	2385	10994	4.61^a^
SEM^4^		13.05	ND	0.03	16.28	ND	0.08	15.70	ND	0.02
Target value^5^		1390	5929	4.27	925	4767	5.15	2315	10696	4.62
Target Value^6^		1390	5880	4.23	885	4585	5.18	2275	10465	4.60
Effect		Probability								
Feed form		<0.001	ND	<0.001	0.37	ND	0.42	0.004	ND	0.004
Nutrient density		0.08	ND	<0.001	0.63	ND	<0.001	0.059	ND	<0.001
Feed form × nutrient density		0.41	ND^7^	0.63	0.90	ND	0.81	0.30	ND	0.41

a,b,c Means in the same column with different superscripts differ significantly (*P* < 0.05).

1 body weight gain

2 feed allocation

3 feed to gain ratio

4 standard error of the means

5 values reported by parent stock Ross 308 manual (Aviagen, 2016)

6 values reported by parent stock Ross 308 manual (Aviagen, 2021).

7 not determined. The star sign shows a significant difference between the treatment with the target values reported by parent stock Ross 308 manual when *P* < 0.05 (*) or *P* < 0.01 (**).

### Body Weight Uniformity and Eating Time

Results of body weight uniformity and eating time of pullets are presented in Table 3. Pullets who were fed crumble or pellet diets had lesser body weight uniformity than those fed mash diet at 7 wk (*P* = 0.014), whereas no differences were observed at 19, 23, and 25 wk of age (*P* > 0.05). Nutrient density did not affect body weight uniformity at 7, 19, 23, and 25 wk of age (*P* > 0.05). The interaction effect between feed form and nutrient density was significant for eating time at 7 wk of age (*P* < 0.001). Eating time in pullets that were fed the mash-diluted diet was longer than pullets fed the mash-standard diet. There was no significant difference between pullets fed the crumble-diluted diet and the pellet-diluted diet, whereas pullets fed the crumble-standard diet had longer eating time rather than those fed the pellet-standard diet. Pullets fed diluted diets had significantly more eating time rather than those fed standard diets at 19 and 23 wk of age (*P* < 0.001). Pullets fed the mash diet spent significantly more eating time than those pullets fed the crumble diet which was longer compared to the pellet diet at 19 as well as 23 wk (*P* < 0.001). A significant interaction effect was observed between feed form and nutrient density for eating time at 25 wk (*P* < 0.001). Diluting the diet led to prolong eating time when pullets were fed the mash diet, whereas this effect was not observed with feeding crumble or pellet diet.

**Table 3:** Influence of feed form and nutrient density on body weight uniformity and eating time in broiler breeder pullets at different ages.

Feed form	Nutrient density	Body weight uniformity (%)				Eating time (min)			
		7 wk	19 wk	23 wk	25 wk	7 wk	19 wk	23 wk	25 wk
Mash	Standard diet	95.44	91.20	89.82	92.97	47.40^b^	32.00	40.80	44.40^b^
Crumble		94.00	89.05	90.77	91.69	29.00^c^	19.60	22.00	25.80^c^
Pellet		93.54	90.37	89.42	91.84	19.80^e^	11.60	12.60	14.80^d^
Mash	Diluted diet	95.05	90.78	92.14	93.13	67.20^a^	38.00	47.60	53.80^a^
Crumble		94.28	90.50	89.88	93.14	28.20d,c	24.60	25.40	28.40^c^
Pellet		94.29	90.25	91.53	93.01	22.80d,e	13.00	14.00	15.40^d^
SEM^1^	0.45	0.77	0.91	0.99	1.25	1.20	1.31	1.40	
Main effect									
Feed form									
Mash		95.25^a^	90.99	90.95	93.05	57.30^a^	35.00^a^	44.20^a^	49.10^a^
Crumble		94.14^b^	89.78	90.33	92.42	28.60^b^	22.10^b^	23.70^b^	27.10^b^
Pellet		93.92^b^	90.31	90.48	92.42	21.30^b^	12.30^c^	13.30^c^	15.10^c^
SEM^1^		0.31	0.54	0.64	0.70	0.89	0.85	0.92	0.99
Nutrient density									
Standard diet		94.33	90.21	90.00	92.17	32.06^b^	21.06^b^	25.13^b^	28.33^b^
Diluted diet		94.54	90.51	91.19	93.10	39.40^a^	25.20^a^	29.00^a^	32.53^a^
SEM^1^		0.26	0.44	0.52	0.57	0.72	0.69	0.75	0.001
Effect	Probability								
Feed form		0.014	0.31	0.12	0.77	<0.001	<0.001	<0.001	<0.001
Nutri density		0.56	0.63	0.76	0.26	<0.001	<0.001	<0.001	<0.001
Feed form × nutrient density		0.45	0.44	0.16	0.79	<0.001	0.154	1.30	0.011

a,b,c,d,e Means in the same column with different superscripts differ significantly (*P* < 0.05).

1 standard error of the means.

### Blood Parameters

The effect of feed form and nutrient density as well as their interaction on blood parameters including cholesterol, glucose, triglyceride, HDL-c, LDL-c, VLDL-c, aspartate transaminase, and alanine transaminase was not significant in breeder pullets at 19 wk (*P* > 0.05, Table 4). Feed form (*P* = 0.007) and nutrient density (*P* = 0.026), not their interaction, affected on H/L ratio in pullets (Table 4). Pullets fed a pellet diet had a greater H/L ratio than those fed crumble or mash diets, and the H/L ratio decreased when the diet was diluted.

**Table 4:** Influence of feed form and nutrient density on blood serum parameters in broiler breeder pullets at 19 wk of age.

Feed form	Nutrient density	Glucose (mg/dL)	TRG^2^ (mg/dL)	Cholesterol (mg/dL)	HDL-c^3^ (mg/dL)	LDL-c^4^ (mg/dL)	VLDL-c^5^ (mg/dL)	AST^6^ (IU/L)	ALT^7^ (IU/L)	H/L^8^
Mash	Standard diet	187.4	106.0	219.4	75.2	123.0	21.2	1335	6.74	0.31
Crumble		200.9	171.0	202.2	84.0	84.0	34.2	1341	7.14	0.27
Pellet		210.2	225.3	230.2	71.8	113.3	45.1	1358	7.36	0.36
Mash	Diluted diet	184.7	138.0	237.0	73.4	136.0	27.6	1366	7.16	0.24
Crumble		208.6	124.7	232.2	72.2	135.1	24.9	1463	8.58	0.25
Pellet		194.5	150.7	205.4	84.2	91.1	30.1	1284	7.64	0.31
SEM^1^		14.49	39.42	20.92	5.38	20.30	7.88	58.94	0.52	0.02
Main effect										
Feed form										
Mash		186.0	122.0	228.2	74.3	129.5	24.4	1351	6.95	0.27^b^
Crumble		204.7	147.8	217.2	78.1	109.5	29.5	1402	7.86	0.26^b^
Pellet		202.0	188.0	217.8	78.0	102.2	37.6	1321	7.50	0.33^a^
SEM^1^		10.25	27.87	14.79	3.80	14.35	5.57	41.68	0.37	0.01
Nutrient density										
Standard diet		199.5	167.4	224.9	77.0	120.7	33.5	1371	7.79	0.31^a^
Diluted diet		195.7	137.8	217.4	76.6	106.8	27.6	1345	7.08	0.27^b^
SEM^1^		8.37	22.76	12.08	3.10	11.72	4.55	34.03	0.30	0.01
Effect		Probability								
Feed form		0.39	0.26	0.84	0.72	0.39	0.26	0.39	0.24	0.007
Nutri density		0.75	0.36	0.66	0.93	0.40	0.36	0.58	0.11	0.026
Feed form × nutrient density		0.71	0.38	0.40	0.099	0.21	0.38	0.26	0.49	0.44

a,b Means in the same column with different superscripts differ significantly (*P* < 0.05).

1 standard error of the means

2 Triglyceride

3 High density lipoprotein-cholesterol

4 Low density lipoprotein-cholesterol

5 Very low density lipoprotein-cholesterol

6 Aspartate transaminase

7 Alanine transaminase

8 Heterophile to lymphocyte ratio.

### Intestinal Traits

The main effect of feed form and nutrient density as well as their interaction on the length and weight of intestines was not statistically significant (*P* > 0.05, Table 5). However, the relative weight of the small intestine was increased numerically by diluting the diets (*P* = 0.06), whereas this effect was not observed for the large intestine.

**Table 5:** Influence of feed form and nutrient density on the absolute length (cm), relative length (cm/% live body weight), and relative weight (% live body weight) of small and large intestines.

Feed form	Nutrient density	Small intestine			Large intestine		
		Absolute length	Relative length	Relative weight	Absolute length	Relative length	Relative weight
Mash	Standard diet	153.4	5.58	1.66	42.5	1.56	0.39
Crumble		154.2	5.52	1.61	42.5	1.52	0.36
Pellet		152.0	5.28	1.48	39.4	1.36	0.38
Mash	Diluted diet	141.4	5.38	1.64	38.8	1.47	0.37
Crumble		157.4	5.72	1.87	40.1	1.45	0.43
Pellet		163.6	5.83	1.77	42.0	1.49	0.42
SEM^1^		5.24	0.22	0.11	1.73	0.06	0.02
Main effect							
Feed form							
Mash		147.4	5.48	1.65	40.7	1.51	0.38
Crumble		155.8	5.56	1.74	41.3	1.49	0.40
Pellet		157.8	5.62	1.62	40.7	1.43	0.40
SEM^1^		3.70	0.15	0.08	1.23	0.04	0.01
Nutrient density							
Standard diet		153.2	5.64	1.58	41.5	1.47	0.37
Diluted diet		154.1	5.46	1.76	40.3	1.47	0.41
SEM^1^		3.02	0.12	0.06	1.00	0.03	0.01
Effect	Probability						
Feed form		0.13	0.81	0.56	0.90	0.48	0.40
Nutri density		0.82	0.31	0.06	0.41	0.97	0.25
Feed form × nutrient density		0.09	0.26	0.35	0.18	0.26	0.16

1 standard error of the means.

## DISCUSSION

### Productive Performance

It is well documented that feeding pelleted diets improves BWG and F:G in broiler chickens (Abdollahi et al., 2018; Mabelebele et al., 2018). However, research on the effect of feed form in laying hens and specifically broiler breeders is limited. Gous and Danisman (2016) found that a diet in mash form is preferred to pellet form to decrease frustration, boredom, hunger, and feather pecking in broiler breeders. Similar to the present experiment, Frikha et al. (2009) showed that feeding a pellet diet and Saldaña et al. (2015) showed that a crumble diet compared to a mash diet improves BWG in egg layer pullets. This improvement seems to be related to the fact that high temperature and pressure used for pelleting or crumbling of the diet change the structure of the nutrients such as starch and protein (Gracia et al., 2009; Zimonja and Svihus, 2009), increases energy (Jiménez-Moreno et al., 2009) and crude protein (Lacassagne et al., 1988) digestibility. In addition, pelleting promotes the access of endogenous enzymes to nutrients (Mateos et al., 2002). In the current study, reducing nutrient density in the diluted diet by using sunflower hulls decreased BWG and increased F:G in pullets. The increasing fiber content of diets results in reducing metabolizable energy which negatively affects the birds' BWG and F:G (Jha and Mishra, 2021). In agreement with the present results, the 4% inclusion of cereal straw or sugar beet pulp as an insoluble fiber source decreased the BWG of the egg layer pullets from 0 to 17 wk of age (Guzmán et al., 2015a). Moreover, Jiménez-Moreno et al. (2013) showed that diluting broiler diet with sugar beet pulp as a fiber source at 2,5%, 5%, and 7.5% decreased the BWG of broiler chickens linearly.

### Body Weight Uniformity and Eating Time

Diet dilution with sunflower hull did not affect body weight uniformity in pullets which is in agreement with the findings of other researchers who fed broiler breeder pullets with fibrous and diluted diets in the rearing period and found no significant effect on uniformity (Zuidhof et al., 2015; de Los Mozos et al., 2017; Asensio et al., 2020). Van Emous et al. (2021) also reported that feeding a low-density diet to broiler breeders (91% at grower and 84% at pre-breeder periods) had no significant effect on body weight uniformity at 10 wk as well as 20 wk of age. Chickens consume a specific amount of a balanced diet in a meal and mixing feed ingredients to prepare a balanced diet is a critical and inventible process in feed manufacturing. The effect of feed (nutrient) uniformity on animal performance has been reviewed by Behnke (1996), who noted that lack of proper mixing can lead to reduced diet uniformity which can affect animal performance.

At 7 wk of age, diluting the mash diet resulted in pullets spending 19.8 min more (67.2 vs. 47.4) eating. Diluting the crumble diet caused pullets to spend 0.8 min (29 vs. 28.2) less eating whereas it was 3 min (22.8 vs. 19.8) more for the pellet-diluted diet. The elevations of eating time at 19, 23, and 25 wk were expected because diet diluting led to the increase in daily feed allocation by roughly 6.8 g (6,314 vs. 5,698 g for 91 d) in the grower, 10.14 g (4,680 vs. 4,254 g for 42 d) in pre-layer, and 7.83 g (10,994 vs. 9,952 g for 133 d) in the entire period of the experiment. Diluting the diets caused pullets to spend 6 min (38 vs. 32) more eating for mash form, 5 min (24.6 vs. 19.6) for crumble, and 1.4 min (13 vs. 11.6) for pellet form at 19 wk, whereas it was 6.8 min (47.6 vs. 40.8), 3.4 min (25.4 vs. 22), and 1.4 min (14 vs. 12.6) for 23 wk, and 9.4 min (53.8 vs. 44.4), 2.6 min (28.4 vs. 25.8), and 0.6 min (15.4 vs. 14.8) for 25 wk, respectively. Similarly, de Los Mozos et al. (2017) reported that 15% diet dilution caused 4 more min eating at 19 wk for broiler breeder pullets fed recommended crumble diet. In a recent study, fibrous diets were fed to broiler breeders and it was found that longer filling of the gastrointestinal tract, might improve a feeling of satiety and decrease stress mainly during the period of rearing (Asensio et al., 2020). Diluting the diets is done with high fiber feeds and feed consumption may be altered depending on the solubility of fiber as Mateos et al. (2012) reported that insoluble fiber gathers in the upper part of the gastrointestinal tract, so fiber increases the retention time of digesta content and can limit nutrient intake of broiler chickens. However, feed intake is different in broiler breeders who are subjected to feed restriction programs from 2 wk onwards to control body weight.

Feed form directly affects eating time, and it was reported that feeding a Pellet diet decreased the eating time and energy consumption of broiler chickens (Savory, 1974). Abdollahi et al. (2013) reported feeding a diet in small particle sizes causes lower retention time in the gastrointestinal tract so that the birds can consume more feed during a specific period. The pelleting process reduces the size of coarse particles and broilers consume more feed with a pellet diet than with a mash diet (Amerah et al., 2007), and hence eating time reduces with pelleting. Bulk density is also an important factor that can influence feed intake as Wan et al. (2021) reported changing mash to pellet form diets causes higher bulk density and improves feed intake therefore eating time decreases with pelleting.

### Blood Parameters

Similar to our results de Jong et al. (2005) reported nutrient density has no significant effect on glucose concentration in broiler breeders during the rearing period. do Vale et al. (2019) also found no significant effect of feeding different sources of fiber to broiler breeders on glycemia.

Broiler breeders are challenged with fasting to control body weight during their life. A review of the literature shows broiler breeders are exposed to this stressful inevitable fasting and appropriate management practices and strategies should be applied to prevent or reduce the effects of stresses (Rosales, 1994). Pullets in this experiment spent roughly between 11 and 67 min eating meaning that they fasted for more than 23 h. Fasting time was longer in pullets fed pellet diet than others and it was also longer in the standard diet than the diluted diet. According to Gross and Siegel (1983), a bigger H/L ratio in pullets fed pellet diets as well as those fed standard diets show that they may be subjected to more chronic stress than others. de Jong et al. (2005) showed that the H/L ratio increases when broiler breeders experience more stress.

### Intestinal Traits

Some researchers have reported that feeding a mash diet increases the length of the gastrointestinal tract in broilers (Nir et al., 1995; Abdollahi et al., 2013, Jiménez-Moreno et al., 2019) and pullets (Frikha et al., 2009). The coarser particles of the mash diets will reduce the feed passage rate from the gizzard to the small intestine, stimulating the contractions, development, and relative weight of the total gastrointestinal tract. However, during pellet processing, dietary ingredients are more finely ground to improve pellet quality, which could lead to increased voluntary feed intake and an overload of digesta in the small intestine (Boazar et al., 2021). We could not find any report about the effect of feed form on the gastrointestinal of broiler breeders. However, it should be considered that broilers are feeding ad-libitum, whereas broiler breeders are restricted to feeding. Therefore, it seems feed form has a lesser effect on gastrointestinal development in broiler breeders.

The small intestine is the main organ in the gastrointestinal tract and its length could support the digestion and absorption of nutrients (Wan et al., 2021). It has been reported that the relative length of the small intestine of broilers fed with a diet that contained fiber sources was higher than the control group (Sittiya et al., 2020), which might be due to the adaptation of the small intestine to allow more feed intake and nutrient deficiency compensation (Mourão et al. 2008). Similarly, Röhe et al. (2020) showed that using different sources of dietary insoluble fiber at different concentrations increased relative digestive tract weights suggesting a fiber-related effect on the development of the intestinal organ. It was stated that an expansion of the digestive tract might be a consequence of physical distension caused by luminal swelling due to consumed fiber sources (Jiménez-Moreno et al., 2013). Enting et al. (2007) reported that diluting diet caused the heavier relative weight of jejunum and ileum in broiler breeders at 22 wk of age because of increasing transport of diet and fermentation, whereas this altering was not observed for cecal and colon. However, the response of poultry to fiber inclusion depends on the source and level of dietary fiber and the characteristics of the diet as well as on the physiological status and health of the bird that has been reviewed by Mateos et al. (2012).

## CONCLUSION

The results of the current experiment are consistent with the hypothesis that pelleting and crumbling the diet increases the growth rate in poultry. Feeding broiler breeder pullets with a pellet diet compared to a mash diet during the rearing period caused increased BWG, faster consumption of feed and ultimately elevated H/L ratio. However, BWG, feeding time, and H/L ratio in pullets fed crumble diet did not differ from those pullets fed mash diet. Diluting pellet and crumble diets with sunflower hull to 90% nutrients recommendations decreased BWG and increased eating time. Hence, diluting pellet or crumble diets with sunflower hull could be considered in broiler breeder pullet feeding with no detrimental effect on their performance or health state in the rearing period.

## DISCLOSURES

The authors declare no conflicts of interest.
